# Resetting of the NEI-RQL-42 scale model for spectacle and contact lens wearers

**DOI:** 10.7717/peerj.21167

**Published:** 2026-04-28

**Authors:** Irene Sanchez, Oscar Garcia Espinilla, Carlos Durantez-Fernández, Itziar Fernández, Sara Ortiz-Toquero, Raul Martin

**Affiliations:** 1 Departamento de Física Teórica, Atómica y Óptica, Universidad de Valladolid, Valladolid, Spain; 2 Optometry Research Group, IOBA Eye Institute, Universidad de Valladolid, Valladolid, Spain; 3 Departamento de Enfermería, Facultad de Enfermería, Universidad de Valladolid, Valladolid, Spain; 4 Nursing Care Research (GICE), Universidad de Valladolid, Valladolid, Spain; 5 Departamento de Estadística e Investigación Operativa, Universidad de Valladolid, Valladolid, Spain

**Keywords:** Contact lens correction, Spectacles correction, NEI-RQL-42

## Abstract

**Background:**

The NEI Refractive Error Quality of Life Instrument 42 (NEI-RQL-42) was developed to assess how refractive error compensation affects quality of life. The purpose of this study was to reset the NEI-RQL-42 scale model to develop specific scales related to the use of ophthalmic lenses and contact lenses (CLs).

**Methods:**

A prospective study was designed. Healthy volunteers between 18 and 70 years of age completed the NEI-RQL-42 questionnaire twice, once for spectacles use and once for CLs use. Confirmatory factor analysis was used to evaluate the original factor and refine the subscales to improve the psychometric properties of the NEI-RQL-42 and detect differences between spectacles and CLs.

**Results:**

Seventy-five healthy subjects (58 women and 17 men with an average age of 30.53 ± 12.51 years) were included. The original factor structure did not show an acceptable fit for spectacles or CLs. Several items were removed from the spectacles and CLs datasets, and some modifications of the subscales based on modification indices were introduced to obtain acceptable models for both samples. Spectacle users showed significantly higher scores (*p* < 0.01) on the clarity of vision, diurnal fluctuations, symptoms and satisfaction with correction subscales. On the other hand, CLs users had higher scores on the “activity limitations” subscale (*p* < 0.01).

**Conclusion:**

analysis of the NEI-RQL-42 score with an adequate statistical approach allows us to identify the differences between the effects of spectacles and CLs wear on patients’ QoL, providing useful information regarding the impact of refractive error compensation on QoL. Our results suggest that it is unlikely that the same scale can be used for different methods of refractive error compensation, such as spectacles or CLs.

## Introduction

Refractive errors such as myopia, hyperopia and astigmatism affect more than 50% of the population (Williams et al., 2015), who must wear spectacles or contact lenses to improve their vision. In 2001, the National Eye Institute developed the NEI Refractive Error Quality of Life Instrument 42 (NEI-RQL-42) (Hays & Spritzer, 2002) to assess how refractive error compensation affects quality of life (QoL) (National Eye Institute, 2002). The NEI-RQL-42 instrument includes 42 multiple response questions (items) subdivided into 13 subscales or domains with 16 different question/response category formats specifically designed for subjects who, through correction of refractive error, have a normal visual acuity (VA) of 20/30 or better (National Eye Institute, 2002; Hays et al., 2003). The use of this instrument is recommended for assessing people who experience symptoms related to their vision and well-being (National Eye Institute, 2002; McAlinden et al., 2011).

The NEI-RQL-42 scale has been used to determine QoL in patients after different surgical options to compensate for refractive errors, including the implantation of different intraocular lens (IOL) designs, (Pedrotti et al., 2018) corneal refractive surgery techniques (Shams et al., 2015) or cataract surgery. (Muttuvelu & Andersen, 2016) Moreover, QoL with contact lens (CLs) compensation has also been studied in some specific populations, such as keratoconus patients, (McAlinden et al., 2012) astigmatism patients who wear toric CLs (Cox et al., 2018), and patients wearing orthokeratology lenses (Lipson, Sugar & Musch, 2005).

The psychometric properties of the NEI-RQL-42 questionnaire have been tested in different languages (Greek (Labiris et al., 2012), French (Djadi-Prat et al., 2011), Turkish (Toker et al., 2008) or Iranian (Pakpour et al., 2013)) and with different refractive error compensation methods (Iijima et al., 2016; McAlinden et al., 2012) suggesting that this scale has adequate psychometric properties to compare QoL in patients with different refractive errors (myopia and hyperopia) (Labiris et al., 2012; Hays et al., 2003) when traditional validation methods are used. However, several deficiencies for all psychometric properties have been reported when QoL was assessed for postrefractive surgery (McAlinden et al., 2011) or keratoconus (Soeters et al., 2018) patients. Although Cronbach’s alpha coefficients seem to be favourable for the use of the NEI-RQL-42 scale, (Djadi-Prat et al., 2011; Pakpour et al., 2013) some Rasch analysis reports suggest (McAlinden et al., 2011; McAlinden et al., 2012) a poor fit that could reflect inherent weaknesses of the original NEI-RQL-42 that may not be related to language translation. The analysis revealed two key issues: several items showed disordered response categories—likely due to an excessive number of response options—and six subscales contained misfitting items, indicating multidimensionality. These problems suggest that parts of the questionnaire are not measuring a single latent trait as intended, raising concerns about the instrument’s validity.

In some cases, the questionnaire indicates that patients experience a decrease in their QoL associated with their refractive error compensation (Shams et al., 2015; Nichols et al., 2003; Queirós et al., 2012). Having an instrument capable of quantifying these changes in a reliable and structured manner would therefore be highly valuable. The NEI-RQL-42 questionnaire was originally developed with this intention, aiming to capture the multidimensional impact of refractive correction in daily life. However, despite its conceptual relevance, its practical use has become restricted to very specific scenarios due to the psychometric limitations described above (McAlinden et al., 2011; McAlinden et al., 2012). As a result, the instrument has not achieved widespread adoption in routine clinical practice or in studies involving the most common forms of refractive correction, such as spectacles and contact lenses, highlighting the need to revisit and refine its structure to ensure its applicability across different compensation methods.

For these reasons, it is proposed to look for new alternatives, as confirmatory factor analysis, to improve the performance of this questionnaire and if possible, reduce the number of items in order to improve the assessment of QoL related to different methods of compensation for refractive errors (such as monofocal, astigmatic (toric) or multifocal ophthalmic lenses; CLs; or refractive surgery) and to detect differences between ophthalmic lenses and CLs wear. This analysis allows us to verify whether the theoretical structure that the questionnaire is intended to measure is reflected in the data obtained. The central idea is to validate that the items adequately represent the dimensions or constructs that are intended to be measured. For these reasons, the aim of this study was to reset the NEI-RQL-42 scale model to develop specific scales to be used for QoL assessment related to the use of ophthalmic lenses and CLs to compensate for refractive errors.

## Materials & Methods

### Subjects

A prospective study involving healthy volunteers aged between 18 and 70 years who used spectacles and CLs to compensate for refractive error was conducted. Exclusion criteria were presence any type of systemic pathology affecting the ocular surface or vision, any ocular surgery and of ocular pathology of any type, especially ocular surface pathology affecting contact lens wear and vision. All subjects completed the NEI-RQL-42 questionnaire twice in the same session, once for spectacle wear and once for CLs wear, in random order. Participants could use any type of correction (spherical, astigmatic, and/or multifocal) as long as they used glasses and any type of contact lenses in combination, without limitation of refractive error. A complete eye exam was carried out to verify the inclusion criteria: corrected monocular and binocular VA better than 20/30 (Snellen scale), use of monofocal or multifocal spectacles and comfortable CLs wear (at least 4 days/week between 4 and 6 h of wear per day) and absence of systemic or any eye pathology that impairs VA. The Human Sciences Ethics Committee of the Valladolid-East Health Area Institutional Review Board granted approval for the study (PI17-532), and written informed consent was obtained from each subject. All subjects were treated in accordance with the Declaration of Helsinki.

### NEI-RQL-42 questionnaire

The NEI-RQL-42 is a 42-item instrument designed for assessing how refractive error compensation affects QoL (National Eye Institute, 2002). According to the manual for use, 42 items are divided into 13 subscales with 16 different question/response category formats and scored on a scale from 0 to 100, where higher scores indicate better QoL (Hays & Spritzer, 2002).

A minimum sample size of 48 subjects was determined to be necessary to detect a minimum difference of two points (margin of error) measured with an alpha risk of 0.05 and a beta risk of 0.05, assuming a standard deviation of seven points to find differences between mean scores of NEI-RQL-42 score. This sample size calculation has been made based on the standard deviations observed in other studies mentioned in the literature. However, the sample size for performing confirmatory factor analysis should be close to or exceed 100 subjects.

The Spanish version of the NEI-RQL-42 was developed in conformance with the international standard methods for the cross-cultural adaptation (Guillemin, Bombardier & Beaton, 1993), including forward translations, construction of a consensus version, back-translation into English (Supplemental Information). Pilot study was performed to assess the translation quality with ten subjects, checking that all subjects had understood all items. Final version was reviewed by five eye care practitioners and was considered appropriate for administration in a psychometric field testing.

### Data analysis

Statistical analysis was performed using R version 3.5.1. The lavaan and semTools packages were used to fit and evaluate the confirmatory factor analysis (CFA) models. Reliability indices were computed using the ltm package (Guillemin, Bombardier & Beaton, 1993).

### Analysis of the factor structure of the NEI-RQL-42

The factor structure (based on the original factors detailed in the NEI-RQL-42 questionnaire manual) of the NEI-RQL-42 was tested using CFA on two datasets, responses for spectacles and CLs. In each dataset, we tested the proposed factor structure followed by changes to the original model based on reliable indices, model fit, and/or modification indices.

Because of the ordinal nature of the item answers, a diagonally weighted least squares procedure was used to estimate the parameters of the CFA models. Model goodness-of-fit was determined by calculating multiple fit indices. First, two absolute fit indices were calculated to determine how well each tested model fit the sample data. To evaluate the overall fit, the chi-square (*χ*^2^) model test was used. A good model fit was not significant at the 0.05 significance level (Dimitris, 2006). To minimize the impact of sample size on the chi-square model statistic, the relative/normed chi-square value, defined as *χ*^2^ statistic/degrees of freedom (Barrett, 2007), was also calculated. In different studies, a value less than 2 (Methodology, 2018) or 5 (Barrett, 2007) could be considered acceptable, so values <2 were chosen as acceptable fitting values for this study. The root-mean-square error of approximation (RMSEA) (Tabachnick & Fidell, 2007) was also calculated with a 90% confidence interval as the absolute index. In a well-fitting model, the RMSEA ranged from 0 to 0.05. The poor fit (RMSEA > 0.05) hypothesis was also tested. Finally, two incremental fit indices, the comparative fit index (CFI) and the nonnormed fit index (NNFI), were calculated to compare the target models with the null/independence model in which all measured variables were uncorrelated (Steiger, 1990). These indices vary from 0 to 1, where values closer to 1 indicate good fit. For both indices, values greater than 0.95 were considered acceptable (Bentler, 1990).

Once the original proposed factor structure was evaluated by CFA, the following aspects were evaluated to refine the subscales and improve the psychometric properties of the NEI-RQL-42 to detect differences between spectacles and CLs wear.

### Item reduction

Interitem correlations were determined using polychoric correlations. According to the recommendations of Hinkin (Hu & Bentler, 1999), any item that correlated at less than 0.4 with all other items of the same subscale was removed from the factor structure. The strength of appropriateness of scoring items together on one subscale was also evaluated by the item-to-total correlation with a polyserial correlation between each individual item and the corresponding score without this item. Item-to-total correlations of 0.3 or more were considered acceptable. Bootstrap 95% confidence intervals based on 1000 resamples were computed for all correlation measures.

### Internal consistency assessment

Cronbach’s alpha coefficients and the corresponding bootstrap 95% (based on 1,000 resamples) confidence intervals were used to evaluate the internal consistency of the subscales. An alpha coefficient of 0.80 or higher was considered an acceptable threshold for reliability. For the subscales with more than two items, the alpha values without each item were computed, and an item was removed if the alpha improved significantly without it.

### *Post hoc* model fit improvement

The *post hoc* modification model approach attempts to improve CFA fit by using modification indices to identify significantly correlated residual error terms. The scales were sequentially improved to determine the model fit according to the following criteria: items with large modification index values (based on a threshold of 4) were included. Items with weak loadings (<0.4) were considered for removal.

### QoL comparison between the type of correction (spectacles *versus* CLs)

After the item reduction process, the Kolmogorov–Smirnov test was performed to determine the normality of the data. Furthermore, subscale scores were normalized to allow comparison, since some subscales of the different questionnaires (spectacles and CLs) included a different number of items. Scores from the 13 subscales of the modified NEI-RQL-42 questionnaire were compared using paired Student’s t tests to determine the differences in QoL between spectacles and CLs wear (*P* values <0.05 were considered to indicate statistical significance).

## Results

### Sample description

A sample of 75 healthy subjects (58 women and 17 men) with an average age of 30.53 ± 12.51 years (median age 25 years and interquartile range 19 years) and an average spherical equivalent refraction of the right eye of −2.47 ± 3.21 diopters (range −14.00 to +8.00) were enrolled in the study. All patients were 18 years or older, were native Spanish speakers and had no cognitive impairment. Of these 75 subjects, 13 used multifocal contact lenses, 15 used toric contact lenses, two used multifocal toric contact lenses and the rest used (45 subjects) spherical contact lenses. Table 1 (Model 0) summarizes the raw responses for the two types of correction (spectacles or CLs) in the original factor structure of the NEI-RQL-42.

**Table 1:** QoL depend on the type of correction. Statistically differences, at 0.05 significance level, between spectacles and CL samples are marked in bold.

**Model 0:** Summary of the scores from 13 sub-scales of original factor structure NEI-RQL-42 questionnaire with spectacles and CL wear compared using paired t-Student test.			
**Score**	**Spectacles sample** **Mean ± SD**	**CL sample** **Mean ± SD**	***p*-value**
**Clarity of vision**	76.39 ± 7.54	60.58 ± 11.02	***p* < 0.01**
**Expectations**	33.33 ± 33.75	46.53 ± 7.26	***p* < 0.01**
**Near vision**	89.36 ± 13.94	67.25 ± 15.47	***p* < 0.01**
**Far vision**	82.94 ± 15.33	81.17 ± 14.71	*p* = 0.31
**Diurnal fluctuations**	88.50 ± 17.81	74.50 ± 17.12	***p* < 0.01**
**Activity limitations**	80.50 ± 22.49	92.17 ± 13.07	***p* < 0.01**
**Glare**	74.83 ± 16.50	44.72 ± 14.65	***p* < 0.01**
**Symptoms**	82.52 ± 8.70	74.57 ± 9.80	***p* < 0.01**
**Dependence on correction**	50.89 ± 22.59	66.06 ± 22.88	***p* < 0.01**
**Worry**	55.67 ± 28.64	61.83 ± 20.44	***p* < 0.01**
**Suboptimal correction**	91.83 ± 20.19	83.83 ± 25.23	***p* < 0.01**
**Appearance**	81.07 ± 23.48	71.16 ± 17.86	***p* < 0.01**
**Satisfaction with correction**	86.67 ± 19.26	82.33 ± 22.13	*p* = 0.13
**Model 3:** Summary of the scores in the modified NEI-RQL-42 questionnaire with spectacles and CL wear compared using paired t-Student test. Higher score means higher satisfaction.			
**Score**	**Spectacles sample** **Mean ± SD**	**CL sample** **Mean ± SD**	***p*-value**
**Clarity of vision**	93.67 ± 17.96	81.67 ± 17.96	**<0.01**
**Expectations**	33.33 ± 33.73	36.67 ± 33.73	0.05
**Near vision**	85.78 ± 20.45	83.25 ± 20.45	0.34
**Far vision**	86.93 ± 16.76	90.22 ± 16.76	0.89
**Diurnal fluctuations**	83.78 ± 25.26	70.00 ± 25.26	**<0.01**
**Activity limitations**	85.56 ± 23.94	95.56 ± 23.94	**<0.01**
**Glare**	88.67 ± 25.10	81.72 ± 25.10	0.05
**Symptoms**	91.13 ± 14.60	63.41 ± 14.60	**<0.01**
**Dependence on correction**	74.00 ± 40.78	72.67 ± 40.78	0.45
**Worry**	55.67 ± 28.64	51.33 ± 28.64	0.07
**Suboptimal correction**	91.44 ± 20.81	86.67 ± 20.81	0.14
**Appearance**	80.53 ± 21.05	–	–
**Satisfaction with correction**	86.67 ± 19.27	79.67 ± 19.27	**<0.01**

**Notes.**

SD, Standard Deviation; CI, Confidence Interval.

### Analysis of the factor structure of the NEI-RQL-42

The evaluation of the original factor structure of the NEI-RQL-42 questionnaire confirmed that there was no acceptable model fit for either sample (Table 2, Model 0), as all the indices were below the expected values. Different modifications were performed to refine the subscales (described below), including item reduction, internal consistency assessment and *post hoc* model fit improvement, as shown in Table 2.

**Table 2:** Fit statistics for confirmatory factor analysis (CFA) models. The initial CFA model (model 0) tests the hypothesized 13-factor structure of the original NEI-RQL-42 questionnaire. Model 1 tests the item reduction modification based on reliable indexes. Model 2 tests the *post hoc* item reduction modification. Model 3 is based on modification indexes in sub-scales after *post hoc* item reduction and internal consistency. Better model values are marked in bold.

Fit statistic		Chi-squared (*df*)	*p*-value	Chi-squared/df	RMSEA (CI 90%)	H_0_: RMSEA ≤ 0.05	CFI	NNFI
Acceptable value		*p* > 0.05		<2	RMSEA < 0.05		>0.95	>0.95
Spectacles sample	Model 0	1,546.9 (*742*)	<0.01	2.08	0.147 (0.137; 0.158)	<0.01	0.43	0.34
	Model 1	708.7 (*418)*	<0.01	1.70	0.116 (0.101; 0.13)	<0.01	0.68	0.59
	Model 2	292.8 (*224*)	<0.01	1.32	0.076 (0.050; 0.099)	0.059	0.89	0.84
	**Model 3**	**250.3 (** ***219*** **)**	**0.07**	**1.14**	**0.051** **(0; 0.079)**	**0.46**	**0.95**	**0.95**
CL sample	Model 0	1,486.6 (*742*)	<0.01	2.00	0.116 (0.107; 0.124)	<0.01	0.55	0.48
	Model 1	852.3 (*529*)	<0.01	1.61	0.09 (0.079; 0.101)	<0.01	0.73	0.68
	Model 2	532.5 (*343*)	<0.01	1.55	0.086 (0.071; 0.1)	0.01	0.78	0.73
	**Model 3**	**346.6 (** ***314*** **)**	**0.10**	**1.10**	**0.037** **(0; 0.059)**	**0.81**	**0.96**	**0.95**

**Notes.**

CL, Contact Lens; df, degree of freedom; RMSEA, Root Mean Square Error of Approximation; CI, Confidence Interval; CFI, Comparative Fix Index; NNFI, Non-Normed Fit Index.

### Item reduction

The first modification of the NEI-RQL-42 questionnaire was based on an initial item reduction, removing items with item–item polychoric correlations lower than 0.4 (absolute value). For the spectacle dataset, items were removed from two subscales, symptoms and far vision. From the symptoms subscale, headache (Item 25) was removed (Table 3A). Moreover, Items 4 and 5, related to the difficulty of judging distances and seeing things off to the side, respectively, were removed from the far vision subscale (Table 3B). For the CLs dataset, Item 27 from the satisfaction of appearance subscale was removed (Table 3C).

**Table 3:** Inter - item polychoric correlations. Correlogram showing the relationship between each pair of items from (A) the “Symptoms” subscale in spectacles dataset; (B) the “Far vision” subscale in spectacles dataset and (C) the “Appearance” subscale in Contact Lens (CL) dataset. Correlation coefficients were colour-coded according to theirs values, negative values are shown in italics. According to the initial item reduction criterion, the items showing correlates below 0.4 (in absolute value) with all other items of the same subscale were considered for removal from the analysis.

(A) ** Symptoms sub-scale**						
	**I19**	**I24**	**I25**	**I36**	**I41**	**I42**
**I18**	0.50	0.64	0.11	0.65	0.61	0.27
	**I19**	0.31	0.31	0.53	0.48	0.21
		**I24**	*−0.04*	0.58	0.70	0.37
			**I25**	0.37	0	0.36
				**I36**	0.72	0.66
					**I41**	0.56
**(B) Far Vision sub-scale**						
			**I5**	**I6**	**I9**	**I10**
		**I4**	0.29	0.33	0.18	0.35
			**I5**	0.12	0.07	0.32
				**I6**	0.46	0.10
					**I9**	0.66
**(C) Appearance sub-scale**						
					**I29**	**I30**
				**I27**	0.34	*−0.18*
					**I29**	*−0.95*

**Figure 1:**
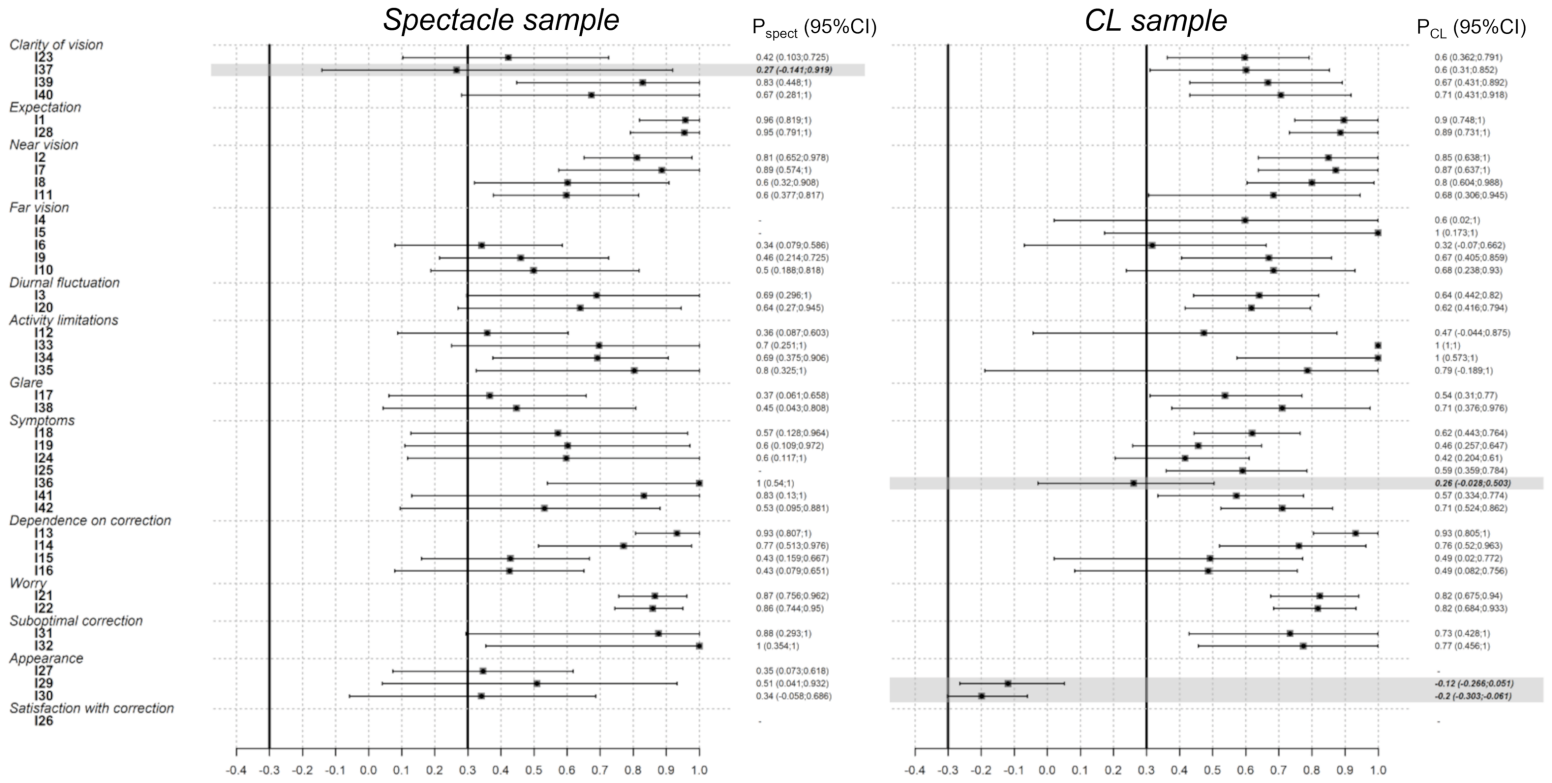
Item-to-total correlations. Polyserial correlation coefficients between each individual item and the total corresponding sub-scale core without that item (r). The 95% bootstrap confidence intervals (CI), based on 1000 resamples, are plotted as horizontal lines. Items that correlate at less than 0.3 (in absolute value) with its sub-scale score will be dropped from the analysis (bold italic text and shadow). The vertical lines represent the no-correlation cut-offs (0.3 and −0.3).

Items with item-to-total correlations lower than 0.30 were also removed from the corresponding subscale. For the spectacle sample, Item 37 (Distorted vision problem) was removed from the clarity of vision subscale (item-to-total correlation of 0.266 (95% CI from −0.141 to 0.919)). For the CLs sample, Item 36 (“tearing”) was removed from the symptoms subscale (item-to-total correlation 0.261; 95% CI from −0.265 to 0.051), and Items 29 (item-to-total correlation −0.119; 95% CI from −0.0285 to 0.503) and 30 (item-to-total correlation −0.198; 95% CI from −0.303 to −0.061) was removed from the appearance subscale, which completely removed this subscale from the CLs sample (Fig. 1).

### Internal consistency assessment

The internal consistency of the subscales was assessed with Cronbach’s alpha coefficients (α). The clarity of vision, far vision, activity limitations, glare, dependence on correction and appearance subscales for spectacles and the activity limitations subscale for CLs showed a value <0.8. Table 4 shows the estimated α values when each item was eliminated. After the elimination of items, the subscales with nonacceptable values of α significantly improved their internal consistency, except for the glare and appearance subscales in the spectacle sample. For the glare subscale, it was not possible to evaluate the reliability without individual items since it is a 2-item subscale. For the appearance subscale, item removal failed to improve the reliability of the subscale. Finally, Item 42 (soreness or tiredness) was removed from the symptom’s subscale in the spectacle sample because despite having a α value significantly greater than 0.8, the reliability of this subscale improved without this item.

**Table 4:** Estimated internal consistency of the NEI-RQL-42 questionnaire sub-scales. Overall Cronbach’s alpha value and the values of alpha when each item is excluded are estimated. In bold are presented alpha coefficient values significantly less than 0.8 (based in 95% CI).

	**Spectacles dataset**		**Contact Lens dataset**	
	**Cronbach’s**α **(95% CI)** **Model 0**	α**without item** **(95% CI)** **Model 1**	**Cronbach’s**α **(95% CI)** **Model 0**	α**without item** **(95% CI)** **Model 1**
**Clarity of vision**	0.642 (0.428; 0.782)	0.742 (0.350; ** 0.952**)	0.715 (0.598; ** 0.808**)	–
**Expectations**	**0.911** (0.824; ** 0.970**)	(*)	**0.876** (0.785; ** 0.942**)	(*)
**Near vision**	0.747 (0.614; ** 0.838**)	–	**0.835** (0.706; ** 0.911**)	–
**Far vision**	0.500 (0.217; 0.707)	0.642 (0.247; ** 0.853**)	0.710 (0.393; ** 0.858**)	0.730 (0.458; ** 0.846**)
**Diurnal fluctuations**	0.664 (0.379; ** 0.828**)	(*)	0.707 (0.526; ** 0.812**)	(*)
**Activity limitations**	0.504 (0.190; 0.708)	0.705 (0.442; ** 0.822**)	0.518 (0.061; 0.705)	0.737 (0.455; ** 0.929**)
**Glare**	0.481 (0.096; 0.727)	(*)	0.649 (0.367; ** 0.810**)	(*)
**Symptoms**	0.681 (0.321; ** 0.830**)	0.712 (0.444; ** 0.846**)	0.751 (0.640; ** 0.823**)	–
**Dependence on correction**	0.678 (0.513; 0.776)	0.711 (0.522; ** 0.821**)	0.680 (0.473; 0.790)	0.723 (0.531; ** 0.837**)
**Worry**	**0.897** (**0.831**; ** 0.949**)	(*)	**0.875** (0.784; ** 0.936**)	(*)
**Suboptimal correction**	0.730 (0.243; ** 0.927**)	(*)	0.739 (0.467; ** 0.908**)	(*)
**Appearance**	0.372 (0.055; 0.586)	–	–	–
**Satisfaction with correction**	(*)	(*)	(*)	(*)

**Notes.**

CI, Confidence Interval.

(*) Cronbach’ α can only be computed in 2 or more items subscales. On 2 items subscales it is no possible to compute α if remove one of them.

### *Post hoc* model fit improvement

The fit statistics for the CFA model revealed slight but insufficient improvement after item reduction (Table 2, Model 1). Therefore, *post hoc* modifications were performed to obtain acceptable CFA models. First, items with a relatively weak factor loading (<0.4) were removed. For the spectacle sample, the following items were removed: items 7 (difficulty reading newspapers) and 8 (difficulty reading small print) from the near vision subscale; Item 17 (see starbursts or halos with bright lights at night”) from the glare subscale; Item 24 (pain or discomfort) from the symptoms subscale; Item 15 (glasses for driving at night) from the dependence on correction subscale; and Items 29 (the type of vision correction you have is now the best you have ever had) and 30 (there is a better type of vision correction) from the appearance subscale (Table 5). For the CLs sample the following items were removed: item 1 (life differences with perfect vision without CLs) from the expectations subscale; Items 4 (difficulty judging distances) and 6 (difficulty getting used to the dark) from the far vision subscale; Item 16 (need to wear CLs for driving at dusk) from the dependence on correction subscale, Item 21 (worry about vision) from the worry subscale; and Item 31 (frequency of type of correction that was uncomfortable) from the suboptimal corrections subscale (Table 6).

**Table 5:** NEI-RQL-42 sub-scales and items in CFA models based on spectacles dataset. Factor loading values in the different models fitted: (i) Model 0: original NEI-RQL-42 questionnaire; (ii) Model 1: item reduction modification based on reliable indices; (iii) Model 2: first *post hoc* modification, removing items with weak factor loading (<0.4); and (iv) Model 3: second *post hoc* modification, adding items sequentially with large values of modification indices (>4).

Sub-scale/item	Factor loadings (*p-value*)			
	Model 0	Model 1	Model 2	Model 3
Clarity of vision				
I23	0.052 (0.654)			
I37	0.229 (0.281)			
I39	0.793 (0.003)	0.761 (0.005)	0.74 (0.007)	0.935 (0.005)
I40	0.614 (0.012)	0.62 (0.008)	0.616 (0.005)	0.656 (0.002)
Expectations				
I1	0.902 (<0.001)	0.98 (<0.001)	0.931 (<0.001)	0.974 (<0.001)
I28	0.936 (<0.001)	0.864 (<0.001)	0.897 (<0.001)	0.867 (<0.001)
Near vision				
I2	0.777 (<0.001)	0.979 (<0.001)	1 (<0.001)	1.081 (<0.001)
I7	0.578 (0.03)	0.37 (0.047)		
I8	0.45 (0.063)	0.328 (0.053)		
I11	0.628 (<0.001)	0.773 (<0.001)	0.759 (0.001)	0.667 (0.002)
Far vision				
I4	0.751 (0.05)			
I5	0.043 (0.853)			
I6	0.498 (0.006)			
I9	0.253 (0.196)	0.667 (0.001)	0.675 (<0.001)	0.75 (<0.001)
I10	0.272 (0.118)	0.71 (<0.001)	0.702 (<0.001)	0.67 (<0.001)
I39				0.498 (0.027)
Diurnal fluctuations				
I3	1.046 (0.001)	0.973 (<0.001)	1.003 (<0.001)	1.233 (<0.001)
I20	0.663 (0.026)	0.688 (0.001)	0.66 (0.003)	0.406 (0.02)
Activity limitations				
I12	0.483 (0.021)			
I33	0.47 (0.01)	0.526 (0.007)	0.507 (0.011)	0.337 (0.166)
I34	0.706 (0.001)	0.77 (<0.001)	0.756 (<0.001)	0.731 (0.001)
I35	0.746 (0.01)	0.978 (<0.001)	0.962 (<0.001)	1.032 (<0.001)
Glare				
I17	0.337 (0.107)	0.39 (0.048)		
I38	1.284 (0.003)	1.075 (0.003)	1.045 (<0.001)	1.045 (<0.001)
Symptoms				
I18	0.541 (0.047)	0.606 (0.01)	0.529 (0.088)	0.423 (0.18)
I19	0.623 (0.002)	0.667 (0.001)	0.664 (0.009)	0.623 (0.011)
I24	0.359 (0.125)	0.383 (0.097)		
I25	0.461 (0.005)			
I36	0.733 (0.006)	0.734 (0.005)	0.718 (0.014)	0.657 (0.024)
I41	0.713 (0.007)	0.683 (0.032)	0.69 (0.032)	0.778 (0.003)
I42	0.556 (<0.001)			
I20				0.573 (<0.001)
Dependence on correction				
I13	1.133 (0.001)	1.529 (0.444)	1.159 (0.017)	1.144 (<0.001)
I14	0.776 (0.003)	0.561 (0.47)	0.724 (0.04)	0.733 (0.002)
I15	0.153 (0.25)	0.141 (0.33)		
I16	0.158 (0.097)			
Worry				
I21	0.826 (0.003)	0.953 (<0.001)	0.885 (<0.001)	0.936 (<0.001)
I22	1.023 (<0.001)	0.897 (<0.001)	0.939 (<0.001)	0.891 (<0.001)
Suboptimal correction				
I31	0.607 (0.001)	0.578 (0.003)	0.561 (0.003)	0.568 (0.002)
I32	0.9 (0.004)	0.915 (0.002)	0.9 (0.002)	0.889 (0.002)
Appearance				
I27	1.588 (0.745)	1.901 (0.585)	0.976 (<0.001)	0.976 (<0.001)
I29	0.246 (0.75)	0.229 (0.632)		
I30	0.11 (0.836)	0.292 (0.425)		
Satisfaction with correction				
I26	1.142 (<0.001)	1.125 (<0.001)	1.109 (<0.001)	1.109 (<0.001)

**Table 6:** NEI-RQL-42 sub-scales and items in CFA models based on contact lens (CL) dataset. Factor loading values in the different models fitted: (i) Model 0: original NEI-RQL-42 questionnaire; (ii) Model 1: item reduction modification based on reliable indices; (iii) Model 2: first *post hoc* modification, removing items with weak factor loading (< 0.4); and (iv) Model 3: second *post hoc* modification, adding items sequentially with large values of modification indices (>4)

Sub-scale/item	Factor loadings (*p-value*)			
	Model 0	Model 1	Model 2	Model 3
Clarity of vision				
I23	0.838 (<0.001)	0.844 (<0.001)	0.83 (<0.001)	0.428 (0.157)
I37	0.47 (<0.001)	0.471 (<0.001)	0.479 (<0.001)	0.582 (<0.001)
I39	0.524 (0.001)	0.52 (0.001)	0.535 (0.001)	0.665 (<0.001)
I40	0.553 (<0.001)	0.544 (<0.001)	0.555 (<0.001)	0.652 (<0.001)
Expectations				
I1	0.11 (0.002)	0.171 (0.361)		
I28	7.072 (<0.001)	4.551 (0.367)	0.993 (<0.001)	0.997 (<0.001)
Near vision				
I2	0.859 (<0.001)	0.86 (<0.001)	0.837 (<0.001)	0.847 (<0.001)
I7	0.666 (<0.001)	0.664 (<0.001)	0.695 (<0.001)	0.656 (<0.001)
I8	0.699 (<0.001)	0.699 (<0.001)	0.729 (<0.001)	0.704 (<0.001)
I11	0.721 (0.001)	0.722 (0.001)	0.71 (0.001)	0.687 (0.001)
Far vision				
I4	0.317 (0.336)	0.333 (0.359)		
I5	0.587 (0.12)	0.594 (0.14)	0.553 (0.081)	0.664 (0.074)
I6	0.194 (0.444)	0.204 (0.472)		
I9	0.822 (<0.001)	0.818 (<0.001)	0.83 (<0.001)	0.597 (<0.001)
I10	0.857 (<0.001)	0.85 (<0.001)	0.889 (<0.001)	0.731 (<0.001)
Diurnal fluctuations				
I3	0.656 (<0.001)	0.657 (<0.001)	0.657 (<0.001)	0.494 (0.133)
I20	0.856 (<0.001)	0.854 (<0.001)	0.855 (<0.001)	0.968 (0.029)
Activity limitations				
I12	0.418 (0.149)			
I33	0.751 (0.064)	0.721 (0.071)	0.711 (0.086)	0.768 (0.053)
I34	0.829 (<0.001)	0.812 (<0.001)	0.85 (<0.001)	0.858 (<0.001)
I35	0.551 (0.147)	0.605 (0.111)	0.572 (0.156)	0.571 (0.066)
Glare				
I17	0.344 (0.296)	0.42 (0.067)	0.441 (0.04)	0.482 (0.089)
I38	1.377 (0.184)	1.129 (0.006)	1.074 (0.002)	1.184 (0.031)
Symptoms				
I18	0.582 (<0.001)	0.576 (<0.001)	0.573 (<0.001)	0.568 (<0.001)
I19	0.502 (<0.001)	0.5 (<0.001)	0.503 (<0.001)	0.55 (<0.001)
I24	0.47 (0.001)	0.479 (0.001)	0.481 (0.001)	0.62 (<0.001)
I25	0.536 (<0.001)	0.523 (<0.001)	0.531 (<0.001)	0.4 (0.001)
I36	0.217 (0.087)			
I41	0.655 (<0.001)	0.659 (<0.001)	0.658 (<0.001)	0.642 (<0.001)
I42	0.795 (<0.001)	0.799 (<0.001)	0.796 (<0.001)	0.814 (<0.001)
Dependence on correction				
I13	0.975 (<0.001)	0.98 (<0.001)	0.958 (<0.001)	0.958 (<0.001)
I14	0.86 (<0.001)	0.856 (<0.001)	0.875 (<0.001)	0.875 (<0.001)
I15	0.177 (0.117)			
I16	0.19 (0.093)	0.185 (0.087)		
Worry				
I21	0.322 (0.718)	0.226 (0.951)		
I22	2.386 (0.711)	3.393 (0.95)	0.993 (<0.001)	0.993 (<0.001)
Suboptimal correction				
I31	0.228 (0.736)	0.323 (0.538)		
I32	2.593 (0.727)	1.826 (0.502)	0.993 (<0.001)	1.034 (<0.001)
Appearance				
I27	0.252 (0.438)			
I29	0.192 (0.409)			
I30	0.05 (0.5)			
Satisfaction with correction				
I26	0.993 (<0.001)	0.993 (<0.001)	0.993 (<0.001)	0.951 (0.023)
I23				0.554 (0.066)

This first *post hoc* modification resulted in an improved model fit for both samples, especially for the spectacle sample (Table 2, Model 2). However, these improvements were insufficient to obtain acceptable goodness-of-fit measures, so modifications of the subscales based on modification indices were introduced. For the spectacle dataset (Table 5), the far Vision and symptoms subscales were modified. Items 39 (blurry vision) and 20 (bothered by changes in clarity) were added to the far vision and symptoms subscales, respectively. For the CLs dataset (Table 6), Item 23 (vision clarity using CLs) was added to the satisfaction with correction subscale. Finally, acceptable models were obtained for both samples (Table 2, Model 3).

### QoL comparison between the type of correction (spectacles *versus* CLs)

Spectacle users exhibited significantly higher scores on the clarity of vision, diurnal fluctuations, symptoms and satisfaction with correction subscales (*p* < 0.01, Table 1, Model 3). On the other hand, the score on the activity limitations subscale was significantly greater in the CLs sample (*p* < 0.01, Table 1, Model 3). Other scores on the border of statistical significance were the expectations subscale, with higher scores among CLs users, and the glare and worry subscales, with higher scores for spectacle users.

## Discussion

While Rasch analysis proved appropriate for evaluating the instrument, the findings indicate that the 13 subscales of the NEI-RQL-42 are inadequate for measuring quality of life related to refractive correction (McAlinden et al., 2011; McAlinden et al., 2012), suggesting that rigorously developed questionnaires that meet standard psychometric properties or different score analyses should be used. However, most of the previous research analysed scores as one dataset combining responses from individuals using spectacles or CLs or after some type of refractive surgery (McAlinden et al., 2011; McAlinden et al., 2012); therefore, to our knowledge, no previous reports have applied CFA to the NEI-RQL-42 to differentiate QoL related to the use of spectacles from that related to CLs wear. CFA has been used with different scales in the low vision population, namely, the NEI VFQ-25 scale and other questionnaires assessing independence (Hinkin, 1998; Marella et al., 2010). Similarly, other questionnaires have studied the impact of macular degeneration or the effectiveness of visual rehabilitation on patients’ QoL (Smith et al., 2019; Marakis et al., 2017). The only scale analysed with CFA for people with normal vision is related to visual fatigue after video display terminal use (Lamoureux et al., 2008; Rajabi-Vardanjani et al., 2014).

Understanding the impact of refractive error compensation on QoL is important in clinical practice because of the different methods of refractive error compensation that are currently available. Some studies have explored this issue, such as Kandel et al. (2017) with a qualitative study in which they identify the main themes related with QoL according with patients’ perception. Other studies developed questionnaires in order to quantify the QoL of people with different refractive corrections. Buckhurst et al. (2012) studied near distance comfort with presbyopia compensation, and Gelsthorpe et al. (2003) used a questionnaire in pre-presbyopic population with different methods to compensate the refractive error. Furthermore, Pesudovs, Garamendi & Elliott (2006) developed a questionnaire to assess CLs wearers QoL (CLIQ). Other scales have been developed only for paediatric population such as The Children’s Vision Function Questionnaire (CVFQ) (Felius et al., 2004) or Student Refractive error and Eyeglasses Questionnaire (SREEQ) (Crescioni et al., 2014). However, this questionnaire (NEI-RQL-42) is not currently used for this purpose, but has been relegated solely to the use of refractive surgery (especially intraocular lenses), (Niazi et al., 2024) keratoconus (Yildiz et al., 2021) or orthokeratology, (Ren et al., 2023) and not for the most common refractive error corrections (glasses and contact lenses not associated with surgery, pathologies or alterations in ocular physiology). To the best of our knowledge, there are no similar jobs to this one.

These results of the NEI-RQL-42 shows that QoL is different depending on the method of refractive error compensation (spectacles or CLs), showing that original NEI-RQL-42 factor structure is not suitable for analysing quality of life with spectacles and/or contact lenses and should be modified.

Analysing the items of each of the 13 subscales, Items 2, 5, 16, 23, 25, 33, 34, and 37 are related only to CLs wear, and Items 1, 27, and 36 are related only to spectacle compensation. This means that some of these items do not contribute information about the trait measurement of interest, as suggested by previous reports (McAlinden et al., 2011; McAlinden et al., 2012). The original NEI-RQL-42 instructions include each item in one single subscale, and previous reports have suggested that subscales are deficient in one or more ways; however, with the model developed here, some items are included in two or more subscales depending on the type of correction (spectacle or CLs) assessed to propose valid subscales with adequate response-category, targeting, person separation and nonmisfitting items. These included Items 5 (lateral vision), 7 and 8 (small print reading), 11 (difficulty in daily activities), 17 (bright lights), 20 (vision fluctuations), 23 (clear vision), 28 (QoL), 33 (sport or outdoor activities), 39 (blurry vision), and 40 (vision troubles). The clarity of vision subscale in the CLs score and the diurnal fluctuations, glare, near vision and satisfaction with correction subscales in the spectacle score were not modified. The appearance subscale was removed from the CLs score, and for the spectacle score, only Item 27 of this subscale remained.

Table 1, Model 3 shows that QoL is greater with spectacles than with CLs in all subscales except for the activity limitations subscale in this sample. The difference between the scores for spectacles and CLs on the clarity of vision subscale was statistically significant (*p* < 0.01) because most of the volunteers used hydrophilic CLs, and this compensation method might not achieve good visual quality in some cases, such as astigmatism. CLs users had significantly (*p* < 0.01) worse scores for the diurnal fluctuations and symptoms subscales than the spectacles users but better scores for the activity limitations subscale (*p* < 0.01); however, for the satisfaction with correction subscale, the difference (*p* < 0.01) favoured spectacle compensation (Rajabi-Vardanjani et al., 2014).

These outcomes and new reduced versions of the NEI-RQL-42 must be validated in another sample (attempted to achieve a more balanced ratio of men to women) with the same patient characteristics to determine the differences between the spectacle and CLs scores. In addition, the sample size for performing a confirmatory factor analysis may be relatively small. Although the results have proven to be robust, their stability in other samples cannot be guaranteed and it cannot be generalized. The low participation of men in this study may also represent a limitation and should be verified in a future sample. Another limitation would be that administering both questionnaires in the same session could potentially introduce a comparison bias. However, as the questions refer to participants’ habitual experiences rather than their situation at a single moment, this effect is expected to be minimal and would not necessarily have been mitigated by conducting two separate visits. Besides, the internal consistency of some subscales was not assessed with Cronbach’s alpha coefficients because the assessment was not possible for 2-item subscales. Overall, the main limitation of this study is the inability to fully validate the proposed scales for spectacles and contact lenses due to the relatively small sample size.

The present study introduces slightly modified scales for spectacles and CLs, capable of identifying differences in specific subscales. These subscales may be useful for monitoring contact lens satisfaction and reducing the risk of CLs drop-outs. However, these scales must be validated in future prospective studies. Furthermore, comparing quality of life with glasses and contact lenses could help determine the moment at which patients consider discontinuing the use of contact lenses. This could be a future application for the scales proposed in this study. Another aspect to consider in futures studies is the refractive error value because some items could be relevant to individuals with high refractive error, such as outdoor activities, night-driving or lateral vision, and further research to explore the impact of the amount of refraction on QoL could be conducted, including a sound statistical analysis.

## Conclusions

In conclusion, analysis of the NEI-RQL-42 score with an adequate statistical approach allows us to identify the differences between the effects of spectacles and CLs wear on patients’ QoL, providing useful information regarding the impact of refractive error compensation on QoL. Our results suggest that it is unlikely that the same scale can be used for different methods of refractive error compensation, such as spectacles or CLs.

## Supplemental Information

10.7717/peerj.21167/supp-1 Supplemental Information 1 Data from the spectacles and contact lens questionnaires

10.7717/peerj.21167/supp-2 Supplemental Information 2 Original questionnaire and shorts questionnaire NEI-RQL-42 for spectacles and contact lenses

10.7717/peerj.21167/supp-3 Supplemental Information 3 Patient instructions

10.7717/peerj.21167/supp-4 Supplemental Information 4 NEI-RQL-42 Manual National Eye Institute Refractive Error Quality of Life Instrument (NEI-RQL-42™), Version 1.0: A Manual for Use and Scoring Ron D. Hays and Karen L. Spritzer February 2002

10.7717/peerj.21167/supp-5 Supplemental Information 5 STROBE checklist

## Additional Information and Declarations

### Data Availability

The following information was supplied regarding data availability: The data from the analysed questionnaires are available in the Supplemental File.
