# Supplementary material for: Resetting of the NEI-RQL-42 scale model for spectacle and contact lens wearers
Source: PeerJ. 2026 Apr 28;14:e21167. doi: 10.7717/peerj.21167 (PMC13134548; doi:10.7717/peerj.21167)
Supplement: Supplemental Information 2 [file peerj-14-21167-s002.docx]

**APPENDIX – 1.- original questionnaire NEI-RQL-42**

**NATIONAL EYE INSTITUTE**

**42-ITEM REFRACTIVE ERROR QUALITY OF LIFE INSTRUMENT**

**Fecha de realización:**

|  |  |  |  |
| --- | --- | --- | --- |

1. Si usted tuviera una visión perfecta sin gafas, lentes de contacto u otro tipo de corrección, ¿Cómo sería de diferente su vida?

***(Marque con una X en la casilla que mejor describa su respuesta.)***

Sin diferencia......................................................... 1
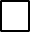


Pequeña diferencia para mejor.................... 2
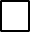


Gran diferencia para mejor............................. 3
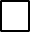


Ya tengo una visión perfecta......................... 4
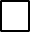


Las siguientes preguntas son acerca del efecto de su visión en sus actividades.

Cuando responda las siguientes cuestiones piense en su visión **CON LA CORRECCIÓN HABITUAL** en gafas y lentes de contacto (en adelante LC) al realizar cada actividad.

1. ¿Cuánta dificultad tiene para realizar trabajos o hobbies que requieren buena visión de cerca, como cocinar, arreglar cosas en casa, costura, utilizar herramientas de mano o trabajar con el ordenador?

***(Marque Una) Gafas LC***

Ninguna dificultad................................................................................................ 1
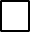


Pequeña dificultad........................................................................................ 2
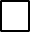


Dificultad moderada ..................................................................................... 3
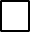


Gran dificultad................................................................................................. 4
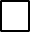


Nunca he intentado hacer estas actividades....................................... 5
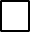


debido a mi visión

Nunca he intentado hacer estas actividades debido ………………….. 6
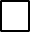


a otras razones.

1. ¿Cuánta dificultad ha tenido debido a cambios de nitidez de su visión a lo largo del día?

***(Marque Una) Gafas LC***

No tengo cambios de nitidez en mi visión .............................................. 1
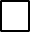


Ninguna dificultad...................................................................................................... 2
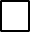


Pequeña dificultad .......................................................................................... 3
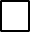


Dificultad moderada........................................................................................ 4
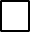


Gran dificultad ................................................................................................... 5
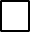


1. ¿Cuánta dificultad tiene para calcular distancias, por ejemplo, bajando escaleras o aparcando el coche?

***(Marque Una) Gafas LC***

Ninguna dificultad....................................................................................................... 1
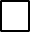


Pequeña dificultad .......................................................................................... 2
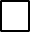


Dificultad moderada........................................................................................ 3
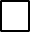


Gran dificultad ................................................................................................... 4
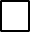


1. ¿Cuánta dificultad tiene para ver cosas laterales, como coches que salen de caminos o calles laterales o personas que salen de portales?

***(Marque Una) Gafas LC***

Ninguna dificultad..................................................................................................... 1
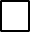


Pequeña dificultad .......................................................................................... 2
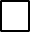


Dificultad moderada........................................................................................ 3
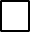


Gran dificultad ................................................................................................... 4
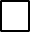


1. ¿Cuánta dificultad tiene adaptarse a la oscuridad cuando pasa de una zona iluminada a un lugar oscuro, como entrar en una sala de cine oscura?

***(Marque Una) Gafas LC***

Ninguna dificultad............................................................................................................ 1
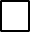


Pequeña dificultad ................................................................................................ 2
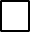


Dificultad moderada.............................................................................................. 3
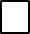


Gran dificultad…………………………………………………………………………………………………..4

1. ¿Cuánta dificultad tiene para leer el periódico impreso?

***(Marque Una) Gafas LC***

Ninguna dificultad..................................................................................................... 1
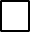


Pequeña dificultad .......................................................................................... 2
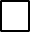


Dificultad moderada........................................................................................ 3
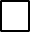


Gran dificultad ................................................................................................... 4
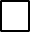


Nunca he intentado hacerlo debido a mi visión................................ 5
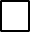


1. ¿Cuánta dificultad tiene para leer letra pequeña como la de la guía telefónica, los prospectos o formularios legales?

***(Marque Una) Gafas LC***

Ninguna dificultad..................................................................................................... 1
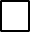


Pequeña dificultad .......................................................................................... 2
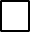


Dificultad moderada........................................................................................ 3
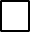


Gran dificultad ................................................................................................... 4
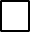


Nunca he intentado hacerlo debido a visión ...................................... 5
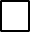


1. ¿Cuánta dificultad tiene para conducir por la noche?

***(Marque Una) Gafas LC***

Ninguna dificultad.................................................................................................... 1
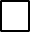


Pequeña dificultad .......................................................................................... 2
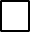


Dificultad moderada ....................................................................................... 3
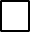


Gran dificultad ................................................................................................... 4
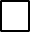


Nunca he intentado hacerlo debido a mi visión............................... 5
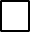


Nunca he intentado hacerlo debido a otras razones ...................... 6
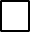


1. ¿Cuánta dificultad tiene conduciendo en condiciones adversas, como por ejemplo, con mal tiempo, en las horas punta, en la autopista o tráfico urbano?

***(Marque Una) Gafas LC***

Ninguna dificultad.................................................................................................... 1
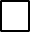


Pequeña dificultad .......................................................................................... 2
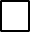


Dificultad moderada....................................................................................... 3
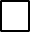


Gran dificultad ................................................................................................... 4
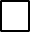


Nunca he intentado hacerlo debido a mi visión............................... 5
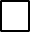


Nunca he intentado hacerlo debido a otras razones ...................... 6
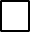


1. Debido a su visión ¿Cuánta dificultad tiene en sus actividades diarias?

***(Marque Una) Gafas LC***

Ninguna dificultad................................................... 1
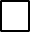


Pequeña dificultad ............................................. 2
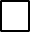


Dificultad moderada.......................................... 3
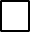


Gran dificultad ...................................................... 4
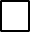


1. Debido a su visión ¿Cuánta dificultad tiene para realizar actividades deportivas u otras actividades al aire libre que le gusten (Por ejemplo: senderismo, natación, aerobic, deportes de equipo o correr)?

***(Marque Una) Gafas LC***

Ninguna dificultad..................................................................................................... 1
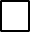


Pequeña dificultad .......................................................................................... 2
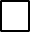


Dificultad moderada....................................................................................... 3
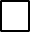


Gran dificultad ................................................................................................... 4
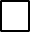


Nunca he intentado hacerlo debido a mi visión.............................. 5
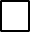


Nunca he intentado hacerlo debido a otras razones ...................... 6
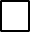


PREGUNTAS SOBRE SU VISIÓN

1. ¿Necesita llevar gafas, bifocales o usar una lupa para leer algo breve como una dirección, un menú o una receta?

***(Marque Una) Gafas LC***

Sí, todo el tiempo............................................................ 1
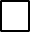


Sí, parte del tiempo....................................................... 2
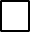


No....................................................................................................................... 3
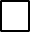


1. ¿Necesita llevar gafas, bifocales o usar una lupa para leer algo extenso, como un libro, un artículo de una revista o un periódico?

***(Marque Una) Gafas LC***

Sí, todo el tiempo............................................................... 1
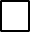


Sí, parte del tiempo.......................................................... 2
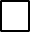


No........................................................................................................................ 3
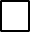


1. Cuando conduce por la noche, ¿Necesita llevar gafas o lentes de contacto?

***(Marque Una) Gafas LC***

Sí, todo el tiempo........................................................................... 1
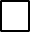


Si, parte del tiempo ...................................................................... 2
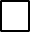


No................................................................................................................... 3
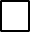


No conduzco por la noche debido a mi vision………….… 4
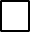


No conduzco por la noche debido a otras razones.…… . 5
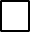


1. Al anochecer, cuando empieza a oscurecer ¿Necesita llevas gafas o lentes de contacto para conducir?

***(Marque Una) Gafas LC***

Sí, todo el tiempo............................................................................... 1
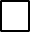


Si, parte del tiempo.......................................................................... 2
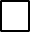


No....................................................................................................................... 3
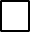


No conduzco por la noche debido a mi visión..................... 4
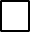


No conduzco por la noche debido a otras razones............ 5
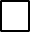


Al responder a estas preguntas piense en su visión con la corrección que habitualmente usa (gafas o lentes de contacto)

1. ¿Con qué frecuencia, por la noche, con las luces brillantes, ve destellos o halos que le molestan o le dificultan la visión?

| ***(Marque Una)*** | ***Gafas*** | ***LC*** |
| --- | --- | --- |
| Todo el tiempo............................................................. | 1 |  |
| La mayor parte del tiempo ................................. | 2 |  |
| Parte del tiempo..................................................... | 3 |  |
| Una pequeña parte del tiempo ........................ | 4 |  |
| Nunca ............................................................................ | 5 |  |

1. ¿Con qué frecuencia experimenta dolor o malestar dentro y alrededor de sus ojos (por ejemplo, picor, ardor o congestión)?

***(Marque Una) Gafas LC***

Todo el tiempo ......................................................... 1
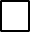


La mayor parte del tiempo .............................. 2
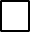


Parte del tiempo................................................... 3
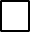


Una pequeña parte del tiempo ................... 4
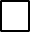


Nunca ............................................................................ 5
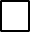


1. ¿Cuánto le molesta la sequedad en los ojos?

***(Marque Una) Gafas LC***

No tengo sequedad ........................ 1
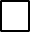


Nada .............................................. 2
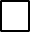


Muy poco ....................................... 3
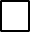


Moderadamente ............................ 4
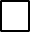


Bastante....................................... 5
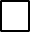


Mucho ......................................... 6
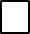


1. ¿Con qué frecuencia le molestan los cambios de nitidez en su visión a lo largo del día?

***(Marque Una) Gafas LC***

Nunca.................................................1
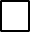


Rara vez..................................................... 2
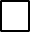


Ocasionalmente.........................3
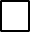


Parte del tiempo ........................4
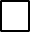


Todo el tiempo .............................5
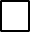


1. ¿Con qué frecuencia se preocupa por su visión?

***(Marque Una) Gafas LC***

Nunca...............................................1
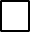


Rara vez................................................... 2
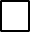


Ocasionalmente .........................3
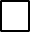


Parte del tiempo.......................4
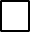


Todo el tiempo ............................5
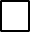


1. ¿Con qué frecuencia se observa o piensa en su vista o visión?

***(Marque Una) Gafas LC***

Nunca ................................................1
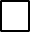


Rara vez...................................................... 2
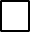


Ocasionalmente ............................3
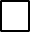


Parte del tiempo .........................4
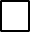


Todo el tiempo ...............................5
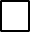


CORRECCIÓN DE SU VISIÓN

Cuando responda a estas preguntas piense en su visión con la corrección habitual con sus gafas y lentes de contacto.

1. En este momento, ¿cómo de clara es su visión utilizando su corrección habitual (gafas o lentes de contacto)?

***(Marque Una) Gafas LC***

Perfectamente clara.................................... 1

Bastante clara................................................. 2

Algo clara...................................................... 3

Nada clara.......................................................... 4

1. ¿Cuánto dolor o malestar experimenta dentro o alrededor de sus ojos (por ejemplo, ardor, picor o congestión)?

***(Marque Una) Gafas LC***

Ninguno....................................................... 1

Leve .................................................2

Moderado....................................... 3

Severo ............................................... 4

Muy severo .................................... 5

1. ¿Con qué frecuencia tiene dolores de cabeza que piensa que están relacionados con su visión o con la corrección de su visión?

***(Marque Una) Gafas LC***

Nunca..................................................................... 1

Rara vez.................................................................... 2

Ocasionalmente.............................................. 3

Parte del tiempo ...................................... 4

Todo el tiempo ................................................ 5

1. ¿Cómo de satisfecho está con su corrección en gafas y lentes de contacto?

***(Marque Una) Gafas LC***

Completamente satisfecho ................................... 1

Muy satisfecho ............................................................. 2

Algo satisfecho .............................................................. 3

Algo insatisfecho....................................................... 4

Muy insatisfecho ......................................................... 5

Completamente insatisfecho................................ 6

1. En cuanto a su apariencia ¿cómo de satisfecho está con sus gafas y lentes de contacto?

***(Marque Una) Gafas LC***

Completamente satisfecho ....................... 1

Muy satisfecho................................................. 2

Algo satisfecho................................................. 3

Algo insatisfecho............................................. 4

Muy insatisfecho ............................................ 5

Completamente insatisfecho.................. 6

1. Si tuviera una visión perfecta sin gafas o lentes de contacto, ¿cuánto cree que cambiaría su vida?

***(Marque Una) Gafas LC***

No cambiaría....................................................... 1

Pequeño cambio a mejor.......................... 2

Gran cambio a mejor ................................ 3

Ya tengo una visión perfecta……......... 4

1. En cuanto a su apariencia, ¿es el tipo de corrección visual que tiene ahora el mejor que ha tenido?

***(Marque Una) Gafas LC***

Si ..................................................... 1

No.............................................2

1. En cuanto a su apariencia, ¿hay algún tipo de corrección visual mejor que el que tiene ahora?

***(Marque Una) Gafas LC***

Si ..................................................... 1

No.............................................2

1. Durante las últimas 4 semanas, ¿con qué frecuencia ha usado un tipo de corrección visual con la que no se sentía cómodo, porque le hacía verse mejor estéticamente?

| ***(Marque Una)*** | ***Gafas*** | ***LC*** |
| --- | --- | --- |
| Todo el tiempo ............................................................. | 1 |  |
| La mayor parte del tiempo ................................... | 2 |  |
| Algunas veces............................................................ | 3 |  |
| Una pequeña parte del tiempo ......................... | 4 |  |
| Nunca ............................................................................... | 5 |  |

1. Durante las últimas 4 semanas, ¿con qué frecuencia ha usado un tipo de corrección visual que no corrige su visión tan bien como otro método porque le hacía verse mejor estéticamente?

| ***(Marque Una)*** | ***Gafas*** | ***LC*** |
| --- | --- | --- |
| Todo el tiempo ............................................................. | 1 |  |
| La mayor parte del tiempo ................................... | 2 |  |
| Algunas veces............................................................ | 3 |  |
| Una pequeña parte del tiempo ......................... | 4 |  |
| Nunca ................................................................................ | 5 |  |

1. Debido a su visión, ¿participa menos de lo que quisiera en actividades deportivas o al aire libre (como senderismo, natación, aerobic, deportes de equipo o salir a correr)?

***(Marque Una) Gafas LC***

Si ...................................................................... 1

No..................................................................2

1. ¿Existe algún tipo de actividad recreativa o deportiva que no realiza debido a su visión o al tipo de corrección visual que utiliza?

***(Marque Una) Gafas LC***

Si, muchas............................................................................1

Si, algunas............................................................................. 2

No..................................................................................................................... 3

1. ¿Hay actividades diarias que le gustaría hacer, pero no las hace debido a su visión o al tipo de corrección visual que utiliza?

***(Marque Una) Gafas LC***

Si, muchas................................................................................1

Si, algunas................................................................................. 2

No ...................................................................................................................... 3

En las últimas 4 semanas, ¿ha tenido alguno de los siguientes problemas? En caso afirmativo,

¿cómo de molesto ha sido? Por favor, responda a cada uno de los problemas, lo haya padecido en uno u ambos ojos.

|  | | ***Marque una*** | | | En caso afirmativo,  ¿cómo de molesto ha sido?  ***(Marque una)*** | |
| --- | --- | --- | --- | --- | --- | --- |
| 36. | ¿Lagrimeo? | **a.** | Gafa  1 | LC | **b.** | Gafa LC  1 |
|  |  | Si.......... |  |  | Mucho.................... |  |
|  |  | No ....... | 2 |  | Algo ................. | 2 |
|  |  |  |  |  | Muy poco | 3 |
|  |  |  |  |  | Nada..................... | 4 |
| 37. | ¿Visión distorsionada? | **a.** | Gafa  1 | LC | **b.** | Gafa LC  1 |
|  |  | Si.......... |  |  | Mucho.................... |  |
|  |  | No ........ | 2 |  | Algo ................. | 2 |
|  |  |  |  |  | Muy poco | 3 |
|  |  |  |  |  | Nada..................... | 4 |
| 38. | ¿Deslumbramiento? | **a.** | Gafa  1 | LC | **b.** | Gafa LC  1 |
|  |  | Si.......... |  |  | Mucho.................... |  |
|  |  | No ........ | 2 |  | Algo ................. | 2 |
|  |  |  |  |  | Muy poco | 3 |
|  |  |  |  |  | Nada..................... | 4 |

En las últimas 4 semanas, ¿ha tenido alguno de los siguientes problemas? En caso afirmativo,

¿cómo de molesto ha sido? Por favor, responda a cada uno de los problemas, lo haya padecido en uno u ambos ojos.

|  | | ***Marque una*** | | | Si es así, ¿Cómo de molesto ha sido?  ***(Marque una)*** | |
| --- | --- | --- | --- | --- | --- | --- |
| 39. | ¿Visión borrosa con el tipo | **a.** | Gafa  1 | LC | **b.** | Gafa LC  1 |
|  | de corrección visual que usa? | Si.......... |  |  | Mucho................. |  |
|  |  | No ........ | 2 |  | Algo................. | 2 |
|  |  |  |  |  | Muy poco | 3 |
|  |  |  |  |  | Nada.................... | 4 |
| 40. | ¿Dificultad para ver? | **a.** | Gafa  1 | LC | **b.** | Gafa LC  1 |
|  |  | Si.......... |  |  | Mucho................. |  |
|  |  | No ........ | 2 |  | Algo................. | 2 |
|  |  |  |  |  | Muy poco | 3 |
|  |  |  |  |  | Nada.................... | 4 |
| 41. | ¿Picor en o alrededor de los ojos? | **a.** | Gafa  1 | LC | **b.** | Gafa LC  1 |
|  |  | Si.......... |  |  | Mucho................. |  |
|  |  | No ......... | 2 |  | Algo................. | 2 |
|  |  |  |  |  | Muy poco | 3 |
|  |  |  |  |  | Nada.................... | 4 |

En las últimas 4 semanas, ¿ha tenido alguno de los siguientes problemas? En caso afirmativo,

¿cómo de molesto ha sido? Por favor, responda a cada uno de los problemas, lo haya padecido en uno u ambos ojos.

|  | | ***Marque una*** | | | Si es así, ¿Cómo de molesto ha sido?  ***(Marque una)*** |
| --- | --- | --- | --- | --- | --- |
| 42. | ¿Dolor o cansancio en | **a.**  Si........ | Gafas  1  2 | LC | **b. Gafa LC** |
|  | los ojos? | No ...... |  |  | Mucho 1 |
|  |  |  |  |  | Algo 2 |
|  |  |  |  |  | Muy poco 3 |
|  |  |  |  |  | Nada 4 |

**APPENDIX B – 2.- short questionnaire NEI-RQL-42 for glasses**

**NATIONAL EYE INSTITUTE**

**42-ITEM REFRACTIVE ERROR QUALITY OF LIFE INSTRUMENT**

**Fecha de realización:**

|  |  |  |  |
| --- | --- | --- | --- |

1. Si usted tuviera una visión perfecta sin gafas, lentes de contacto u otro tipo de corrección, ¿Cómo sería de diferente su vida?

***(Marque con una X en la casilla que mejor describa su respuesta.)***

Sin diferencia......................................................... 1

Pequeña diferencia para mejor.................... 2

Gran diferencia para mejor............................. 3

Ya tengo una visión perfecta......................... 4

Las siguientes preguntas son acerca del efecto de su visión en sus actividades.

Cuando responda las siguientes cuestiones piense en su visión **CON LA CORRECCIÓN HABITUAL** en gafas y lentes de contacto (en adelante LC) al realizar cada actividad.

1. ¿Cuánta dificultad tiene para realizar trabajos o hobbies que requieren buena visión de cerca, como cocinar, arreglar cosas en casa, costura, utilizar herramientas de mano o trabajar con el ordenador?

***(Marque Una) Gafas LC***

Ninguna dificultad................................................................................................ 1

Pequeña dificultad........................................................................................ 2

Dificultad moderada ..................................................................................... 3

Gran dificultad................................................................................................. 4

Nunca he intentado hacer estas actividades....................................... 5

debido a mi visión

Nunca he intentado hacer estas actividades debido ………………….. 6

a otras razones.

1. ¿Cuánta dificultad ha tenido debido a cambios de nitidez de su visión a lo largo del día?

***(Marque Una) Gafas LC***

No tengo cambios de nitidez en mi visión .............................................. 1

Ninguna dificultad...................................................................................................... 2

Pequeña dificultad .......................................................................................... 3

Dificultad moderada........................................................................................ 4

Gran dificultad ................................................................................................... 5

1. ¿Cuánta dificultad tiene para conducir por la noche?

***(Marque Una) Gafas LC***

Ninguna dificultad.................................................................................................... 1

Pequeña dificultad .......................................................................................... 2

Dificultad moderada ....................................................................................... 3

Gran dificultad ................................................................................................... 4

Nunca he intentado hacerlo debido a mi visión............................... 5

Nunca he intentado hacerlo debido a otras razones ...................... 6

1. ¿Cuánta dificultad tiene conduciendo en condiciones adversas, como por ejemplo, con mal tiempo, en las horas punta, en la autopista o tráfico urbano?

***(Marque Una) Gafas LC***

Ninguna dificultad.................................................................................................... 1

Pequeña dificultad .......................................................................................... 2

Dificultad moderada....................................................................................... 3

Gran dificultad ................................................................................................... 4

Nunca he intentado hacerlo debido a mi visión............................... 5

Nunca he intentado hacerlo debido a otras razones ...................... 6

1. Debido a su visión ¿Cuánta dificultad tiene en sus actividades diarias?

***(Marque Una) Gafas LC***

Ninguna dificultad................................................... 1

Pequeña dificultad ............................................. 2

Dificultad moderada.......................................... 3

Gran dificultad ...................................................... 4

PREGUNTAS SOBRE SU VISIÓN

1. ¿Necesita llevar gafas, bifocales o usar una lupa para leer algo breve como una dirección, un menú o una receta?

***(Marque Una) Gafas LC***

Sí, todo el tiempo............................................................ 1

Sí, parte del tiempo....................................................... 2

No....................................................................................................................... 3

1. ¿Necesita llevar gafas, bifocales o usar una lupa para leer algo extenso, como un libro, un artículo de una revista o un periódico?

***(Marque Una) Gafas LC***

Sí, todo el tiempo............................................................... 1

Sí, parte del tiempo.......................................................... 2

No........................................................................................................................ 3

Al responder a estas preguntas piense en su visión con la corrección que habitualmente usa (gafas o lentes de contacto)

1. ¿Con qué frecuencia experimenta dolor o malestar dentro y alrededor de sus ojos (por ejemplo, picor, ardor o congestión)?

***(Marque Una) Gafas LC***

Todo el tiempo ......................................................... 1

La mayor parte del tiempo .............................. 2

Parte del tiempo................................................... 3

Una pequeña parte del tiempo ................... 4

Nunca ............................................................................ 5

1. ¿Cuánto le molesta la sequedad en los ojos?

***(Marque Una) Gafas LC***

No tengo sequedad ........................ 1

Nada .............................................. 2

Muy poco ....................................... 3

Moderadamente ............................ 4

Bastante....................................... 5

Mucho ......................................... 6

1. ¿Con qué frecuencia le molestan los cambios de nitidez en su visión a lo largo del día?

***(Marque Una) Gafas LC***

Nunca.................................................1

Rara vez..................................................... 2

Ocasionalmente.........................3

Parte del tiempo ........................4

Todo el tiempo .............................5

1. ¿Con qué frecuencia se preocupa por su visión?

***(Marque Una) Gafas LC***

Nunca...............................................1

Rara vez................................................... 2

Ocasionalmente .........................3

Parte del tiempo.......................4

Todo el tiempo ............................5

1. ¿Con qué frecuencia se observa o piensa en su vista o visión?

***(Marque Una) Gafas LC***

Nunca ................................................1

Rara vez...................................................... 2

Ocasionalmente ............................3

Parte del tiempo .........................4

Todo el tiempo ...............................5

CORRECCIÓN DE SU VISIÓN

Cuando responda a estas preguntas piense en su visión con la corrección habitual con sus gafas y lentes de contacto.

1. ¿Cómo de satisfecho está con su corrección en gafas y lentes de contacto?

***(Marque Una) Gafas LC***

Completamente satisfecho ................................... 1

Muy satisfecho ............................................................. 2

Algo satisfecho .............................................................. 3

Algo insatisfecho....................................................... 4

Muy insatisfecho ......................................................... 5

Completamente insatisfecho................................ 6

1. En cuanto a su apariencia ¿cómo de satisfecho está con sus gafas y lentes de contacto?

***(Marque Una) Gafas LC***

Completamente satisfecho ....................... 1

Muy satisfecho................................................. 2

Algo satisfecho................................................. 3

Algo insatisfecho............................................. 4

Muy insatisfecho ............................................ 5

Completamente insatisfecho.................. 6

1. Si tuviera una visión perfecta sin gafas o lentes de contacto, ¿cuánto cree que cambiaría su vida?

***(Marque Una) Gafas LC***

No cambiaría....................................................... 1

Pequeño cambio a mejor.......................... 2

Gran cambio a mejor ................................ 3

Ya tengo una visión perfecta……......... 4

1. Durante las últimas 4 semanas, ¿con qué frecuencia ha usado un tipo de corrección visual con la que no se sentía cómodo, porque le hacía verse mejor estéticamente?

| ***(Marque Una)*** | ***Gafas*** | ***LC*** |
| --- | --- | --- |
| Todo el tiempo ............................................................. | 1 |  |
| La mayor parte del tiempo ................................... | 2 |  |
| Algunas veces............................................................ | 3 |  |
| Una pequeña parte del tiempo ......................... | 4 |  |
| Nunca ............................................................................... | 5 |  |

|  |  |  |
| --- | --- | --- |

1. Durante las últimas 4 semanas, ¿con qué frecuencia ha usado un tipo de corrección visual que no corrige su visión tan bien como otro método porque le hacía verse mejor estéticamente?

| ***(Marque Una)*** | ***Gafas*** | ***LC*** |
| --- | --- | --- |
| Todo el tiempo ............................................................. | 1 |  |
| La mayor parte del tiempo ................................... | 2 |  |
| Algunas veces............................................................ | 3 |  |
| Una pequeña parte del tiempo ......................... | 4 |  |
| Nunca ................................................................................ | 5 |  |

1. Debido a su visión, ¿participa menos de lo que quisiera en actividades deportivas o al aire libre (como senderismo, natación, aerobic, deportes de equipo o salir a correr)?

***(Marque Una) Gafas LC***

Si ...................................................................... 1

No..................................................................2

1. ¿Existe algún tipo de actividad recreativa o deportiva que no realiza debido a su visión o al tipo de corrección visual que utiliza?

***(Marque Una) Gafas LC***

Si, muchas............................................................................1

Si, algunas............................................................................. 2

No..................................................................................................................... 3

1. ¿Hay actividades diarias que le gustaría hacer, pero no las hace debido a su visión o al tipo de corrección visual que utiliza?

***(Marque Una) Gafas LC***

Si, muchas................................................................................1

Si, algunas................................................................................. 2

No ...................................................................................................................... 3

En las últimas 4 semanas, ¿ha tenido alguno de los siguientes problemas? En caso afirmativo,

¿cómo de molesto ha sido? Por favor, responda a cada uno de los problemas, lo haya padecido en uno u ambos ojos.

|  | | ***Marque una*** | | | En caso afirmativo,  ¿cómo de molesto ha sido?  ***(Marque una)*** | |
| --- | --- | --- | --- | --- | --- | --- |
| 22. | ¿Lagrimeo? | **a.** | Gafa  1 | LC | **b.** | Gafa LC  1 |
|  |  | Si.......... |  |  | Mucho.................... |  |
|  |  | No ....... | 2 |  | Algo ................. | 2 |
|  |  |  |  |  | Muy poco | 3 |
|  |  |  |  |  | Nada..................... | 4 |
| 23. | ¿Deslumbramiento? | **a.** | Gafa  1 | LC | **b.** | Gafa LC  1 |
|  |  | Si.......... |  |  | Mucho.................... |  |
|  |  | No ........ | 2 |  | Algo ................. | 2 |
|  |  |  |  |  | Muy poco | 3 |
|  |  |  |  |  | Nada..................... | 4 |

En las últimas 4 semanas, ¿ha tenido alguno de los siguientes problemas? En caso afirmativo,

¿cómo de molesto ha sido? Por favor, responda a cada uno de los problemas, lo haya padecido en uno u ambos ojos.

|  | | ***Marque una*** | | | Si es así, ¿Cómo de molesto ha sido?  ***(Marque una)*** | |
| --- | --- | --- | --- | --- | --- | --- |
| 24. | ¿Visión borrosa con el tipo | **a.** | Gafa  1 | LC | **b.** | Gafa LC  1 |
|  | de corrección visual que usa? | Si.......... |  |  | Mucho................. |  |
|  |  | No ........ | 2 |  | Algo................. | 2 |
|  |  |  |  |  | Muy poco | 3 |
|  |  |  |  |  | Nada.................... | 4 |
| 25. | ¿Dificultad para ver? | **a.** | Gafa  1 | LC | **b.** | Gafa LC  1 |
|  |  | Si.......... |  |  | Mucho................. |  |
|  |  | No ........ | 2 |  | Algo................. | 2 |
|  |  |  |  |  | Muy poco | 3 |
|  |  |  |  |  | Nada.................... | 4 |
| 26. | ¿Picor en o alrededor de los ojos? | **a.** | Gafa  1 | LC | **b.** | Gafa LC  1 |
|  |  | Si.......... |  |  | Mucho................. |  |
|  |  | No ......... | 2 |  | Algo................. | 2 |
|  |  |  |  |  | Muy poco | 3 |
|  |  |  |  |  | Nada.................... | 4 |

**APPENDIX B – 3.- short questionnaire NEI-RQL-42 for contact lenses**

**NATIONAL EYE INSTITUTE**

**42-ITEM REFRACTIVE ERROR QUALITY OF LIFE INSTRUMENT**

**Fecha de realización:**

|  |  |  |  |
| --- | --- | --- | --- |

Las siguientes preguntas son acerca del efecto de su visión en sus actividades.

Cuando responda las siguientes cuestiones piense en su visión **CON LA CORRECCIÓN HABITUAL** en gafas y lentes de contacto (en adelante LC) al realizar cada actividad.

1. ¿Cuánta dificultad tiene para realizar trabajos o hobbies que requieren buena visión de cerca, como cocinar, arreglar cosas en casa, costura, utilizar herramientas de mano o trabajar con el ordenador?

***(Marque Una) Gafas LC***

Ninguna dificultad................................................................................................ 1

Pequeña dificultad........................................................................................ 2

Dificultad moderada ..................................................................................... 3

Gran dificultad................................................................................................. 4

Nunca he intentado hacer estas actividades....................................... 5

debido a mi visión

Nunca he intentado hacer estas actividades debido ………………….. 6

a otras razones.

1. ¿Cuánta dificultad ha tenido debido a cambios de nitidez de su visión a lo largo del día?

***(Marque Una) Gafas LC***

No tengo cambios de nitidez en mi visión .............................................. 1

Ninguna dificultad...................................................................................................... 2

Pequeña dificultad .......................................................................................... 3

Dificultad moderada........................................................................................ 4

Gran dificultad ................................................................................................... 5

1. ¿Cuánta dificultad tiene para ver cosas laterales, como coches que salen de caminos o calles laterales o personas que salen de portales?

***(Marque Una) Gafas LC***

Ninguna dificultad..................................................................................................... 1

Pequeña dificultad .......................................................................................... 2

Dificultad moderada........................................................................................ 3

Gran dificultad ................................................................................................... 4

1. ¿Cuánta dificultad tiene para leer el periódico impreso?

***(Marque Una) Gafas LC***

Ninguna dificultad..................................................................................................... 1

Pequeña dificultad .......................................................................................... 2

Dificultad moderada........................................................................................ 3

Gran dificultad ................................................................................................... 4

Nunca he intentado hacerlo debido a mi visión................................ 5

1. ¿Cuánta dificultad tiene para leer letra pequeña como la de la guía telefónica, los prospectos o formularios legales?

***(Marque Una) Gafas LC***

Ninguna dificultad..................................................................................................... 1

Pequeña dificultad .......................................................................................... 2

Dificultad moderada........................................................................................ 3

Gran dificultad ................................................................................................... 4

Nunca he intentado hacerlo debido a visión ...................................... 5

1. ¿Cuánta dificultad tiene para conducir por la noche?

***(Marque Una) Gafas LC***

Ninguna dificultad.................................................................................................... 1

Pequeña dificultad .......................................................................................... 2

Dificultad moderada ....................................................................................... 3

Gran dificultad ................................................................................................... 4

Nunca he intentado hacerlo debido a mi visión............................... 5

Nunca he intentado hacerlo debido a otras razones ...................... 6

¿Cuánta dificultad tiene conduciendo en condiciones adversas, como por ejemplo, con mal tiempo, en las horas punta, en la autopista o tráfico urbano?

***(Marque Una) Gafas LC***

Ninguna dificultad.................................................................................................... 1

Pequeña dificultad .......................................................................................... 2

Dificultad moderada....................................................................................... 3

Gran dificultad ................................................................................................... 4

Nunca he intentado hacerlo debido a mi visión............................... 5

Nunca he intentado hacerlo debido a otras razones ...................... 6

1. Debido a su visión ¿Cuánta dificultad tiene en sus actividades diarias?

***(Marque Una) Gafas LC***

Ninguna dificultad................................................... 1

Pequeña dificultad ............................................. 2

Dificultad moderada.......................................... 3

Gran dificultad ...................................................... 4

PREGUNTAS SOBRE SU VISIÓN

1. ¿Necesita llevar gafas, bifocales o usar una lupa para leer algo breve como una dirección, un menú o una receta?

***(Marque Una) Gafas LC***

Sí, todo el tiempo............................................................ 1

Sí, parte del tiempo....................................................... 2

No....................................................................................................................... 3

1. ¿Necesita llevar gafas, bifocales o usar una lupa para leer algo extenso, como un libro, un artículo de una revista o un periódico?

***(Marque Una) Gafas LC***

Sí, todo el tiempo............................................................... 1

Sí, parte del tiempo.......................................................... 2

No........................................................................................................................ 3

Al responder a estas preguntas piense en su visión con la corrección que habitualmente usa (gafas o lentes de contacto)

1. ¿Con qué frecuencia, por la noche, con las luces brillantes, ve destellos o halos que le molestan o le dificultan la visión?

| ***(Marque Una)*** | ***Gafas*** | ***LC*** |
| --- | --- | --- |
| Todo el tiempo............................................................. | 1 |  |
| La mayor parte del tiempo ................................. | 2 |  |
| Parte del tiempo..................................................... | 3 |  |
| Una pequeña parte del tiempo ........................ | 4 |  |
| Nunca ............................................................................ | 5 |  |

1. ¿Con qué frecuencia experimenta dolor o malestar dentro y alrededor de sus ojos (por ejemplo, picor, ardor o congestión)?

***(Marque Una) Gafas LC***

Todo el tiempo ......................................................... 1

La mayor parte del tiempo .............................. 2

Parte del tiempo................................................... 3

Una pequeña parte del tiempo ................... 4

Nunca ............................................................................ 5

1. ¿Cuánto le molesta la sequedad en los ojos?

***(Marque Una) Gafas LC***

No tengo sequedad ........................ 1

Nada .............................................. 2

Muy poco ....................................... 3

Moderadamente ............................ 4

Bastante....................................... 5

Mucho ......................................... 6

1. ¿Con qué frecuencia le molestan los cambios de nitidez en su visión a lo largo del día?

***(Marque Una) Gafas LC***

Nunca.................................................1

Rara vez..................................................... 2

Ocasionalmente.........................3

Parte del tiempo ........................4

Todo el tiempo .............................5

1. ¿Con qué frecuencia se observa o piensa en su vista o visión?

***(Marque Una) Gafas LC***

Nunca ................................................1

Rara vez...................................................... 2

Ocasionalmente ............................3

Parte del tiempo .........................4

Todo el tiempo ...............................5

CORRECCIÓN DE SU VISIÓN

Cuando responda a estas preguntas piense en su visión con la corrección habitual con sus gafas y lentes de contacto.

1. En este momento, ¿cómo de clara es su visión utilizando su corrección habitual (gafas o lentes de contacto)?

***(Marque Una) Gafas LC***

Perfectamente clara.................................... 1

Bastante clara................................................. 2

Algo clara...................................................... 3

Nada clara.......................................................... 4

1. ¿Cuánto dolor o malestar experimenta dentro o alrededor de sus ojos (por ejemplo, ardor, picor o congestión)?

***(Marque Una) Gafas LC***

Ninguno....................................................... 1

Leve .................................................2

Moderado....................................... 3

Severo ............................................... 4

Muy severo .................................... 5

1. ¿Con qué frecuencia tiene dolores de cabeza que piensa que están relacionados con su visión o con la corrección de su visión?

***(Marque Una) Gafas LC***

Nunca..................................................................... 1

Rara vez.................................................................... 2

Ocasionalmente.............................................. 3

Parte del tiempo ...................................... 4

Todo el tiempo ................................................ 5

1. ¿Cómo de satisfecho está con su corrección en gafas y lentes de contacto?

***(Marque Una) Gafas LC***

Completamente satisfecho ................................... 1

Muy satisfecho ............................................................. 2

Algo satisfecho .............................................................. 3

Algo insatisfecho....................................................... 4

Muy insatisfecho ......................................................... 5

Completamente insatisfecho................................ 6

1. Si tuviera una visión perfecta sin gafas o lentes de contacto, ¿cuánto cree que cambiaría su vida?

***(Marque Una) Gafas LC***

No cambiaría....................................................... 1

Pequeño cambio a mejor.......................... 2

Gran cambio a mejor ................................ 3

Ya tengo una visión perfecta……......... 4

1. Durante las últimas 4 semanas, ¿con qué frecuencia ha usado un tipo de corrección visual que no corrige su visión tan bien como otro método porque le hacía verse mejor estéticamente?

| ***(Marque Una)*** | ***Gafas*** | ***LC*** |
| --- | --- | --- |
| Todo el tiempo ............................................................. | 1 |  |
| La mayor parte del tiempo ................................... | 2 |  |
| Algunas veces............................................................ | 3 |  |
| Una pequeña parte del tiempo ......................... | 4 |  |
| Nunca ................................................................................ | 5 |  |

1. Debido a su visión, ¿participa menos de lo que quisiera en actividades deportivas o al aire libre (como senderismo, natación, aerobic, deportes de equipo o salir a correr)?

***(Marque Una) Gafas LC***

Si ...................................................................... 1

No..................................................................2

1. ¿Existe algún tipo de actividad recreativa o deportiva que no realiza debido a su visión o al tipo de corrección visual que utiliza?

***(Marque Una) Gafas LC***

Si, muchas............................................................................1

Si, algunas............................................................................. 2

No..................................................................................................................... 3

1. ¿Hay actividades diarias que le gustaría hacer, pero no las hace debido a su visión o al tipo de corrección visual que utiliza?

***(Marque Una) Gafas LC***

Si, muchas................................................................................1

Si, algunas................................................................................. 2

No ...................................................................................................................... 3

En las últimas 4 semanas, ¿ha tenido alguno de los siguientes problemas? En caso afirmativo,

¿cómo de molesto ha sido? Por favor, responda a cada uno de los problemas, lo haya padecido en uno u ambos ojos.

|  | | ***Marque una*** | | | En caso afirmativo,  ¿cómo de molesto ha sido?  ***(Marque una)*** | |
| --- | --- | --- | --- | --- | --- | --- |
| 24. | ¿Visión distorsionada? | **a.** | Gafa  1 | LC | **b.** | Gafa LC  1 |
|  |  | Si.......... |  |  | Mucho.................... |  |
|  |  | No ........ | 2 |  | Algo ................. | 2 |
|  |  |  |  |  | Muy poco | 3 |
|  |  |  |  |  | Nada..................... | 4 |
| 25. | ¿Deslumbramiento? | **a.** | Gafa  1 | LC | **b.** | Gafa LC  1 |
|  |  | Si.......... |  |  | Mucho.................... |  |
|  |  | No ........ | 2 |  | Algo ................. | 2 |
|  |  |  |  |  | Muy poco | 3 |
|  |  |  |  |  | Nada..................... | 4 |

En las últimas 4 semanas, ¿ha tenido alguno de los siguientes problemas? En caso afirmativo,

¿cómo de molesto ha sido? Por favor, responda a cada uno de los problemas, lo haya padecido en uno u ambos ojos.

|  | | ***Marque una*** | | | Si es así, ¿Cómo de molesto ha sido?  ***(Marque una)*** | |
| --- | --- | --- | --- | --- | --- | --- |
| 26. | ¿Visión borrosa con el tipo | **a.** | Gafa  1 | LC | **b.** | Gafa LC  1 |
|  | de corrección visual que usa? | Si.......... |  |  | Mucho................. |  |
|  |  | No ........ | 2 |  | Algo................. | 2 |
|  |  |  |  |  | Muy poco | 3 |
|  |  |  |  |  | Nada.................... | 4 |
| 27. | ¿Dificultad para ver? | **a.** | Gafa  1 | LC | **b.** | Gafa LC  1 |
|  |  | Si.......... |  |  | Mucho................. |  |
|  |  | No ........ | 2 |  | Algo................. | 2 |
|  |  |  |  |  | Muy poco | 3 |
|  |  |  |  |  | Nada.................... | 4 |
| 28. | ¿Picor en o alrededor de los ojos? | **a.** | Gafa  1 | LC | **b.** | Gafa LC  1 |
|  |  | Si.......... |  |  | Mucho................. |  |
|  |  | No ......... | 2 |  | Algo................. | 2 |
|  |  |  |  |  | Muy poco | 3 |
|  |  |  |  |  | Nada.................... | 4 |

En las últimas 4 semanas, ¿ha tenido alguno de los siguientes problemas? En caso afirmativo,

¿cómo de molesto ha sido? Por favor, responda a cada uno de los problemas, lo haya padecido en uno u ambos ojos.

|  | | ***Marque una*** | | | Si es así, ¿Cómo de molesto ha sido?  ***(Marque una)*** |
| --- | --- | --- | --- | --- | --- |
| 29. | ¿Dolor o cansancio en | **a.**  Si........ | Gafas  1  2 | LC | **b. Gafa LC** |
|  | los ojos? | No ...... |  |  | Mucho 1 |
|  |  |  |  |  | Algo 2 |
|  |  |  |  |  | Muy poco 3 |
|  |  |  |  |  | Nada 4 |
