# Supplementary material for: Resetting of the NEI-RQL-42 scale model for spectacle and contact lens wearers
Source: PeerJ. 2026 Apr 28;14:e21167. doi: 10.7717/peerj.21167 (PMC13134548; doi:10.7717/peerj.21167)
Supplement: Supplemental Information 3 [file peerj-14-21167-s003.pdf]

Instrucciones:

A continuación realizaremos un cuestionario con las posibles situaciones acerca de los problemas que afectan a su calidad visual o sentimientos que tenga acerca de la corrección de su defecto visual. Después de cada pregunta, por favor seleccione la respuesta que mejor describa su situación.

Por favor, tómese el tiempo que usted necesite en contestar cada pregunta. Todas sus respuestas son confidenciales. El propósito de este cuestionario es mejorar nuestro conocimiento sobre la corrección de la visión y cómo afecta a su vida, por lo que sus respuestas deben ser lo más precisas posible.

1.- Nos gustaría que rellene las respuestas a estas preguntas por sí mismo, si es posible.

2.- Por favor, conteste todas las preguntas (a menos que se le pida que se salte alguna pregunta, ya que no se aplique a usted).

3.- Contesta las preguntas marcando la casilla correspondiente a su respuesta.

4.- Si no está seguro de cómo responder a una pregunta, por favor dé la mejor respuesta que pueda y hacer un comentario en el margen izquierdo.

5. Por favor, complete el cuestionario antes de abandonar el centro y déselo a un miembro del personal del proyecto. No se lo lleve a casa.

DECLARACIÓN DE CONFIDENCIALIDAD:

Toda la información que permita la identificación de cualquier persona que ha completado este cuestionario será considerada como estrictamente confidencial. Dicha información se utilizará únicamente para los fines de este estudio y no será compartida o divulgada para cualquier otro propósito sin el consentimiento previo, excepto cuando sea requerido por la ley.
