# Supplementary material for: Resetting of the NEI-RQL-42 scale model for spectacle and contact lens wearers
Source: PeerJ. 2026 Apr 28;14:e21167. doi: 10.7717/peerj.21167 (PMC13134548; doi:10.7717/peerj.21167)
Supplement: Supplemental Information 4 — National Eye Institute Refractive Error Quality of Life Instrument (NEI-RQL-42™), Version 1.0: A Manual for Use and Scoring Ron D. Hays and Karen L. Spritzer February 2002 [file peerj-14-21167-s004.pdf]

National Eye Institute Refractive Error Quality of Life Instrument (NEI-RQL-42™), Version 1.0:

A Manual for Use and Scoring

Ron D. Hays and Karen L. Spritzer
February 2002

Note that the following citation is suggested when referencing this manual:

Hays, R. D., & Spritzer, K. L. (2002, February). National Eye Institute Refractive Error Quality of Life Instrument (NEI-RQL-42™), Version 1.0: A Manual for Use and Scoring. Los Angeles, CA.

SCORING RULES

Scoring the RQL-42 is a two-step process:

- First, original numeric values from the survey are recoded following the scoring rules outlined in Table 1. All items are scored so that a high score represents better quality of life. Each item is then converted to a 0 to 100 possible range so that the lowest and highest possible scores are set at 0 and 100, respectively. In this format, scores represent the achieved percentage of the total possible score. For example, a score of 50 represents 50% of the highest possible score.

- Second, items within each scale are averaged together to create the 13 scale scores. Table 2 indicates which items contribute to each scale. Scales with at least one item answered can be used to generate a scale score. Items that are left blank (missing data) are not taken into account when calculating the scale scores. Scores represent the average for all items in the scale that the respondent answered.

Copyright © RAND 2002

Table 1. Scoring Key: Recoding of Items

ITEM NUMBERS	Original response category	To recoded value of
1, 28	1 ---->	100
	2 ---->	50
	3 ---->	0
	4 ---->	100
2, 9, 10, 12	1 ---->	100
	2 ---->	75
	3 ---->	50
	4 ---->	25
	5 ---->	0
	6 ---->	*
3	1 ---->	100
	2 ---->	100
	3 ---->	200/3
	4 ---->	100/3
	5 ---->	0
4, 5, 6, 11, 23	1 ---->	100
	2 ---->	200/3
	3 ---->	100/3
	4 ---->	0
7, 8, 20, 21, 22, 24, 25	1 ---->	100
	2 ---->	75
	3 ---->	50
	4 ---->	25
	5 ---->	0
13, 14, 34, 35	1 ---->	0
	2 ---->	50
	3 ---->	100
15, 16	1 ---->	100/3
	2 ---->	200/3
	3 ---->	100
	4 ---->	0
	5 ---->	*
17, 18, 31, 32	1 ---->	0
	2 ---->	25
	3 ---->	50
	4 ---->	75
	5 ---->	100
19	1 ---->	100
	2 ---->	100
	3 ---->	75
	4 ---->	50
	5 ---->	25
	6 ---->	0

* Response choice indicates that the person does not perform the activity because of non-vision related problems. If this choice is selected, the item is coded as “missing.”

Table 1. Scoring Key: Recoding of Items (continued)

ITEM NUMBERS	Original response category	To recoded value of
26, 27	1 ---->	100
	2 ---->	80
	3 ---->	60
	4 ---->	40
	5 ---->	20
	6 ---->	0
29	1 ---->	100
	2 ---->	0
30, 33	1 ---->	0
	2 ---->	100
36b[(†)], 37b[(†)], 38b[(†)], 39b[(†)], 40b[(†)], 41b[(†)], 42b[(†)]	(b=1) ---->	0
	(b=2) ---->	25
	(b=3) ---->	50
	(b=4) ---->	75
	(a=2 and b=missing) ---->	100

[†] Items 36b-42b have four response levels, but are expanded to five levels using items 36a-42a, respectively. If a = 2, then b should have been left blank. If there is a discrepancy between a and b, the user needs to decide how to resolve the discrepancy. In many cases, going with the response to b (ignoring a) when there is a discrepancy may be reasonable.

Table 2: Averaging Items to Generate RQL-42 Scales

Scale	Number of Items	After Recoding Per Table 1, Average the Following Items
Clarity of vision	4	23, 37b, 39b, 40b
Expectations	2	1, 28
Near vision	4	2, 7, 8, 11
Far vision	5	4, 5, 6, 9, 10
Diurnal fluctuations	2	3, 20
Activity limitations	4	12, 33, 34, 35
Glare	2	17, 38b
Symptoms	7	18, 19, 24, 25, 36b, 41b, 42b
Dependence on correction	4	13, 14, 15, 16
Worry	2	21, 22
Suboptimal correction	2	31, 32
Appearance	3	27, 29, 30
Satisfaction with correction	1	26

Table 3: Central Tendency, variability (including floor and ceiling effects), and reliability of RQL-42 Scales‡

Measure	Mean	Standard Deviation	% Floor	% Ceiling	Internal Consistency Reliability
Clarity of vision	83.85	18.36	0.1	27.3	0.72
Expectations	43.57	38.22	34.6	22.2	0.90
Near vision	83.94	18.03	0.0	33.5	0.85
Far vision	83.48	15.85	0.0	20.0	0.81
Diurnal fluctuations	74.58	23.13	0.3	30.3	0.73
Activity limitations	85.28	21.92	0.1	53.5	0.76
Glare	76.40	26.41	1.6	40.1	o.75
Symptoms	79.20	16.79	0.0	12.7	0.78
Dependence on correction	42.38	34.75	28.6	15.2	0.74
Worry	61.31	26.04	3.6	10.1	0.80
Suboptimal correction	92.74	17.28	0.8	81.5	0.64
Appearance	79.31	27.00	0.7	31.8	0.66
Satisfaction with correction	74.85	22.55	1.5	28.4	NA

‡ Data is from a cross-sectional study consisting of 665 myopes, 375 hyperopes, and 114 emmetropes recruited from the practices of six medical centers.

NA – Not applicable for a single-item measure.

 Copyright © RAND 2002
